# Chondroitin Sulfate for Cartilage Regeneration, Administered Topically Using a Nanostructured Formulation

**DOI:** 10.3390/ijms251810023

**Published:** 2024-09-18

**Authors:** Marta E. Bustos Araya, Anna Nardi-Ricart, Ana C. Calpena Capmany, Montserrat Miñarro Carmona

**Affiliations:** 1 Instituto de Investigaciones Farmacéuticas, Facultad de Farmacia, Universidad de Costa Rica, San José 11501, Costa Rica; marta.bustosaraya@ucr.ac.cr; 2 Pharmacy, Pharmaceutical Technology and Physico-Chemical Department, Universitat de Barcelona, Av. Joan XXIII, 27–31, 08028 Barcelona, Spain; annanardi@ub.edu (A.N.-R.); minarromontse@ub.edu (M.M.C.); 3 Institute of Nanoscience and Nanotechnology (IN2UB), University of Barcelona, 08028 Barcelona, Spain; 4 IDIBELL-UB Research Group, Pharmacotherapy, Pharmacogenomics and Pharmaceutical Technology, Avinguda Granvia, 199–203, 08908 L’Hospitalet de Llobregat, Spain

**Keywords:** solid lipid nanoparticles, chondroitin sulfate, osteoarthritis, skin permeation, design of experiments, cell viability, biopharmaceutical studies, drug delivery

## Abstract

In the pharmaceutical sector, solid lipid nanoparticles (SLN) are vital for drug delivery incorporating a lipid core. Chondroitin sulfate (CHON) is crucial for cartilage health. It is often used in osteoarthritis (OA) treatment. Due to conflicting results from clinical trials on CHON’s efficacy in OA treatment, there has been a shift toward exploring effective topical systems utilizing nanotechnology. This study aimed to optimize a solid lipid nanoparticle formulation aiming to enhance CHON permeation for OA therapy. A 3 × 3 × 2 Design of these experiments determined the ideal parameters: a CHON concentration of 0.4 mg/mL, operating at 20,000 rpm speed, and processing for 10 min for SLN production. Transmission electron microscopy analysis confirmed the nanoparticles’ spherical morphology, ensuring crucial uniformity for efficient drug delivery. Cell viability assessments showed no significant cytotoxicity within the tested parameters, indicating a safe profile for potential clinical application. The cell internalization assay indicates successful internalization at 1.5 h and 24 h post-treatment. Biopharmaceutical studies supported SLNs, indicating them to be effective CHON carriers through the skin, showcasing improved skin permeation and CHON retention compared to conventional methods. In summary, this study successfully optimized SLN formulation for efficient CHON transport through pig ear skin with no cellular toxicity, highlighting SLNs’ potential as promising carriers to enhance CHON delivery in OA treatment and advance nanotechnology-based therapeutic strategies in pharmaceutical formulations.

## 1. Introduction

In the pharmaceutical industry, there are a great variety of pharmaceutical forms, depending on the drug used and its pharmaceutical application. At present, nanotechnology is becoming more widespread in many fields. The use of nanoparticles as a vehicle for the transport of active substances can result in the modification of the contact surface, an increase in the permeability of biological barriers, resistance to chemical degradation, the administration of several substances together, or the stimulation of the biological response [1].

Nanoparticles can be greatly diverse in terms of composition, functionality, and characteristics. The uses and applications of each type of nanoparticle will depend both on the physicochemical properties of the drugs (namely, aqueous solubility, oil/water partition coefficient, ionization constant, and stability at various pH prevalent in the gastrointestinal tract), as well as on their characteristic biopharmaceutical properties and their permeability across cell barriers, tissue distribution, cellular uptake, metabolic and excretion patterns, route of drug administration, and general pharmacodynamics and the adverse effects they create [2].

The development of nanostructured lipid carriers and solid lipid nanoparticles dates back to the early 1990s [3]. These types of nanoparticles are described as colloidal solid particles that range in size from 1 nm to 1000 nm. They are made up of lipids that have the ability to disperse in solutions such as water and act as dosing agents in drug delivery [4,5]. Lipid nanoparticles include solid lipid nanoparticles (SLN), nanostructured lipid carriers (NLC), lipid drug conjugate (LDC), and polymer–lipid hybrid nanoparticles (PLN). All this means that these entities emerged as a pharmaceutical alternative to liposomes and emulsions.

SLN are heterogeneous systems that are solids at room and body temperatures, with a lipid inner phase and an external aqueous phase stabilized by surfactants. They are composed of three main components: lipids; surfactants; and the active ingredient (drug) loaded [6].

The solid inner matrix allows for the controlled release of encapsulated molecules and protects them from degradation, increasing their long-term stability. In contrast, the external phase produces interactions with the skin and helps to dissolve nanoparticles. These characteristics present several advantages for cutaneous use, such as protection of the encapsulated molecules from degradation due to the solid matrix, which leads to the possibility that the modulating molecules are released and adhere to the horny layer and penetrate deeper layers due to the hydrophilic interactions between the surfactant of the external layer and lipids present in the stratum corneum [7,8,9].

Solid lipid nanoparticles integrate the advantages of liposomes, emulsions, and polymeric nanoparticles [10,11]. They offer the stability of a solid matrix while also ensuring the biological compatibility of lipid carriers, thus overcoming the drawbacks associated with these drug delivery systems. The solid matrix in SLNs helps protect encapsulated drugs from chemical degradation and facilitates their controlled release. Compared to liposomal systems, SLNs can maintain their stability for longer periods. The components of SLNs are generally well-tolerated, classified, and recognized by and large as safe (GRAS), as they significantly reduce cytotoxicity—a characteristic often lacking in polymeric nanoparticles. This low cytotoxicity expands the potential applications of SLNs to various routes, including dermal, oral, pulmonary, and parenteral administration [12,13,14]. Additionally, SLNs do not exhibit issues such as unpredictable particle growth or unforeseen drug release from the lipid core, which are common challenges in other systems [15].

In general, solid lipid nanoparticles have the following potential advantages: they offer a target-based drug delivery system; drugs are released for a prolonged time; they are less expensive; they are suitable when used for poorly water-soluble drugs; they have long-term stability; they can be administered by different routes, and the carrier does not exhibit bio-toxicity [5,16,17].

Chondroitin sulfate (CHON) is an important biomolecule, which is present in human and animal cartilage. It is commonly ingested orally to treat osteoarthritis (OA), either alone or in conjunction with glucosamine or other ingredients.

Osteoarthritis is a widely occurring Rheumatic Musculoskeletal Disorder, affecting approximately 303 million people worldwide as of 2017 [18,19]. While this disorder can impact any joint where articular cartilage is present, it most commonly affects the knees, hands, hips, and spine [20]. Articular cartilage, a key connective tissue essential for joint function and movement [21], is a viscoelastic structure composed of chondrocytes embedded within a complex extracellular matrix containing collagens, proteoglycans, and glycoproteins [22]. Its complex architecture and limited capacity for self-repair (due to its lack of blood vessels and low cell regeneration) make it vulnerable to chondral injuries [23,24].

These localized areas of damage may result from acute or repetitive trauma, aging, genetic conditions, or other joint-related diseases. If left untreated, chondral injuries can lead to chronic pain, joint dysfunction, inflammation, and the early development of osteoarthritis, all of which significantly diminish the quality of life. OA greatly and detrimentally impacts individuals by causing pain and disability and imposes a substantial economic burden on both patients and society. In 2016, this heavy disease burden prompted the Osteoarthritis Research Society International (OARSI) to submit a white paper, officially recognizing osteoarthritis as a serious disease [25].

As it has low oral bioavailability, studies are exploring modifications of the molecular weight of CHON, which will require further research so as to determine the effectiveness in OA of the modified CHON when administered orally. In this disease, the cartilage in the joints breaks down, and the consumption of chondroitin sulfate may delay this. 

Usually made from animal sources, such as porcine, bovine, and shark cartilage, it can also be produced in the laboratory [26,27].

CHON presents characteristics such as a size of between 5 and 50 kDa. It is the most abundant glycosaminoglycan in the human body, and it is found in cartilage, tendons, ligaments, and the aorta. CHON binds to proteins (like collagen) to form proteoglycan aggregates [28].

Chondroitin sulfate, together with glucosamine sulfate, are two drugs that have the approval of the Spanish Agency for Medicines and Health Products as symptomatic treatment for osteoarthritis. However, their efficiency and safety, which has already been demonstrated through meta-analysis and clinical trials, were questioned in another meta-analysis published by the British Medical Journal (2010), which additionally pointed out that these two drugs did not provide or imply an advantage at the therapeutic level compared to other older drugs [29].

The controversy regarding the therapeutic efficiency of CHON for patients with OA stems from the results evidenced in clinical trials carried out (due to its reduced oral bioavailability and its action as an active ingredient when administered orally), leading to the recommendation to use effective topical systems through nanotechnology as an alternative that allows for the therapeutic action of CHON to be efficient [30,31].

As a result of the need to generate new ways of administering CHON and the advantages that SLNs have shown as transporters of various molecules, the main objective of the present study is the development of an optimized formulation of solid lipid nanoparticles as a vehicle of CHON for skin permeation in OA treatment.

In order to obtain an optimal formulation, different studies have been carried out, such as an experimental factorial design, the development of analytical methods for the determination of the encapsulation percentage of the biomolecule, cellular internalization studies, skin permeation tests, and toxicity trials.

## 2. Results and Discussion

### 2.1. Results of Factorial Design

In accordance with the design of experiments (DOE) 3 × 3 × 2, with three factors at three and two levels, respectively, 18 combinations were established to study the proposed factors. The average of three measurements for each experiment carried out in duplicate is reported in Table 1.

Once the results of all the formulations proposed in the DOE had been obtained, the statistical analysis of the data was carried out. This was to find the formulation with the best characteristics in terms of size, Z potential, and efficiency in CHON encapsulation. In the Supplementary Materials, a more detailed presentation of the raw data for the data analysis evaluation of the design of experiments (DOE) is provided. This includes an analysis of variance and two-way interactions, and the outcomes of these analyses are illustrated in Figures S1–S4.

**Figure 1:**
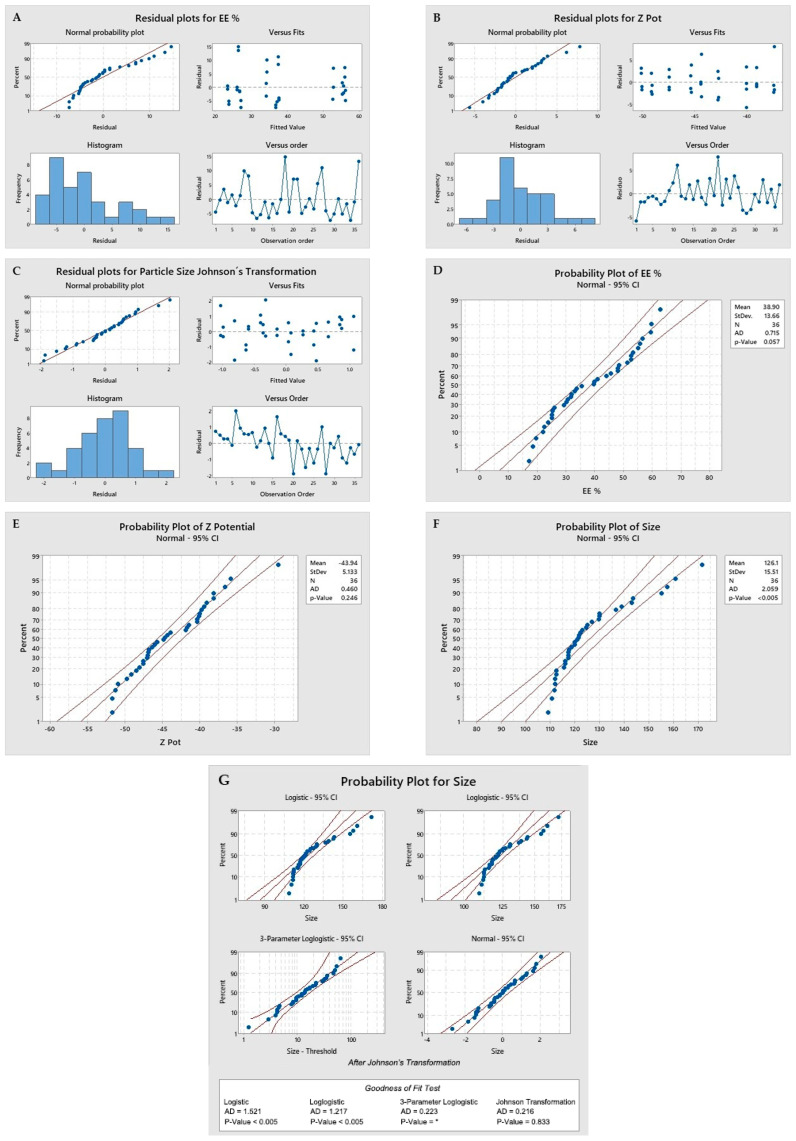
Plots of the DOE premises evaluation. Graphs (**A**–**C**) are used to evaluate the normality of the residuals, linearity, independence, and homoscedasticity. Graphs (**D**–**G**) help to assess the normality of the data, with a value of *p* ≥ 0.05.

First, the statistical premises (normality of residues, linearity, homoscedasticity, and independence) were evaluated (Figure 1).

As seen in the above figure, the following statistical premises for the proposed model are met:

- Normality of residuals and linearity: It is shown when the residuals are aligned to the red line in the upper left box of images A, B, and C. Images D, E, and F show a *p*-value greater than 0.05, indicating a normal distribution. In the case of the evaluation of the size values, a Johnson transformation was required. Images F and G, for which the transformed data were used in the statistical results, are reported in Table 1;
- Homoscedasticity: The fitted value of the residuals shows values above and below zero, clearly seen in the second box at the top right of images A, B, and C;
- Independence: When evaluating the residuals versus order of observation, in the boxes at the bottom right of images A, B, and C. As no patterns are observed in the distribution of the residuals either above or below zero, there is independence.

**Figure 2:**
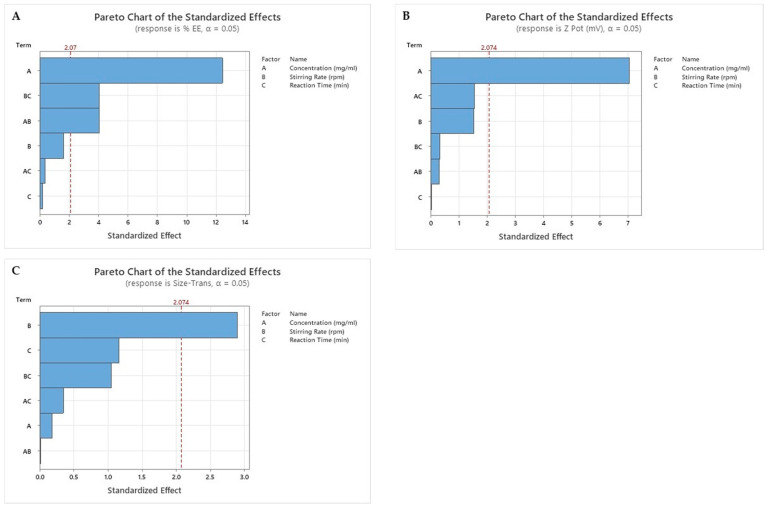
Pareto chart of main effects in DOE. % EE, Z potential and particle size, are represented in (**A**–**C**) respectively. Bars that cross the reference line are statistically significant at the 0.05 level with the current model terms. The main effects that are statistically significant (α = 0.05) are those values that exceed the intermittent vertical red line. The null hypothesis is that the coefficient of the term is equal to zero, which implies that there is no association between the term and the response.

This model met the statistical premises, so each factor proposed in the formulation was evaluated. This study of the impact of the factors on the results of the EE%, particle size, and Zeta Potential was evaluated using a Pareto Chart (Figure 2).

*p-*value ≤ *α:* The association is statistically significant. 

If the *p*-value is less than or equal to the significance level, there is a statistically significant association between the response variable and the term.

*p-*value > *α:* The association is not statistically significant.

If the *p*-value is greater than the significance level, it cannot be concluded that there is a statistically significant association between the response variable and the term.

The EE% is mainly influenced by the concentration of CHON. However, the combination of factors such as the stirring rate with the concentration and the stirring rate with the reaction time have an important effect, and their role should be considered in the production of SLN (Figure 2A). Only the factor concentration is statistically significant for the zeta potential (Figure 2B). The stirring rate is statistically significant for the particle size response (Figure 2C).

**Figure 3:**
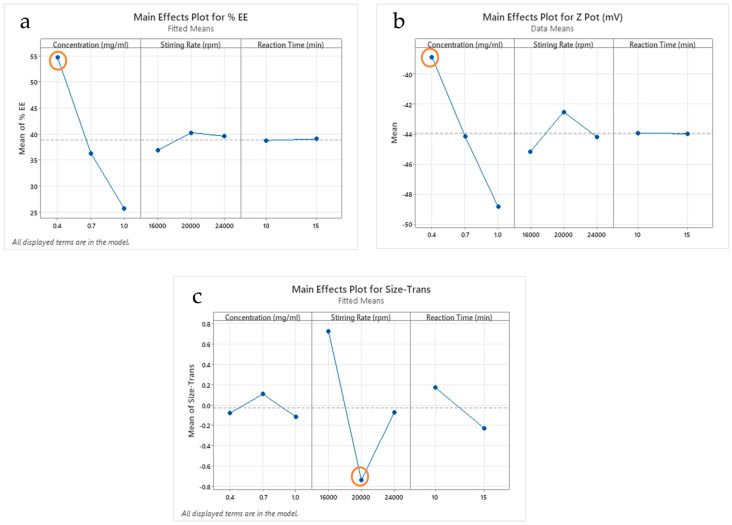
Graph of the main effects, according to the factors and levels defined in DOE.

To determine which level had a greater impact on the results, a plot of the main effects was used, as shown in Figure 3.

The concentration level of 0.4 mg/mL is statistically significant in that it produces the highest EE%, around 55%. The stirring rate of 20,000 rpm is the main effect for EE%. The reaction time is not statistically significant (Figure 3a). Likewise, the 0.4 mg/mL concentration of CHON is the main effect for Zeta potential, with values around −40 mV. 

The stirring factors and the reaction time do not significantly influence the response (Figure 3b). The particle size shows no statistically significant changes for the different levels of concentration and reaction time. However, the stirring factor of 20,000 rpm is statistically significant in obtaining a particle size less than 120 nm (Figure 3c). The two-way ANOVA results are statistically significant at the 0.05 level with the current model term. 

Once the DOE is finished, the best conditions are established: use a CHON concentration of 0.4 mg/mL, a speed of 20,000 rpm, and a time of 10 min for the SLN manufacturing process. The summary of the optimal SLNs that were obtained is shown in Table 2.

**Figure 4:**
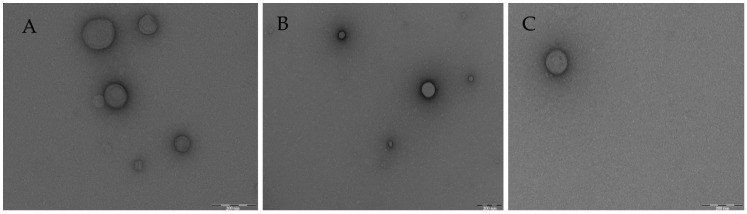
TEM images of SLN with CHON in aqueous medium as part of DOE study. (**A**) 0.4 mg/mL of CHON; (**B**) 0.7 mg/mL of CHON, and (**C**) 1.0 mg/mL of CHON. Scale bar  =  200 nm.

The morphology of the nanoparticles analyzed through transmission electron microscopy (TEM) reveals that the nanoparticles show spherical morphologies, and the observed particle size remained around 150 nm with an average diameter for the three concentrations studied. These findings confirm the average scores obtained for the formulations manufactured at the DOE (Figure 4).

The other DOE data can be seen in Supplementary Materials, where the best-fit equations and all the DOE details are included, together with the progressive exclusion of variables.

### 2.2. Cell Viability; Cytotoxicity

To assess the viability of the cells in the presence of the nanoparticles, the cell cytotoxicity assay with 3-(4,5-dimethylthiazol-2-yl)-2,5-diphenyltetrazolium bromide (MTT) was performed [32]. This study was carried out in human keratinocyte cells (HACAT).

This type of assay precedes others that are required, such as cell internalization and cell transfection, among others. It is necessary to know the concentration or the number of nanoparticles that can be added to a specific number of cells and then achieve the studied effect without causing cellular cytotoxicity. 

**Figure 5:**
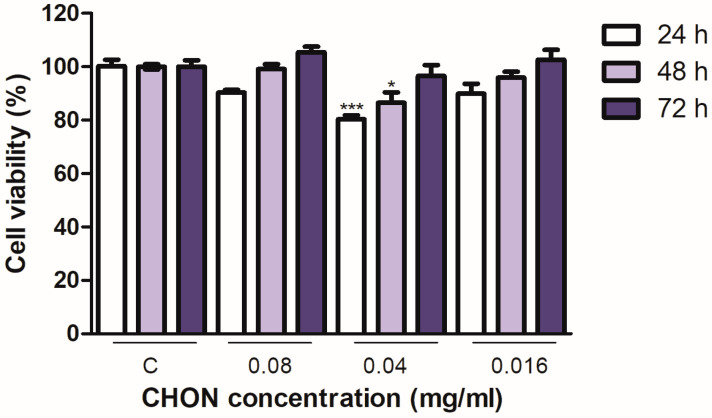
Effect of SLN on the viability of HaCaT cells (keratinocyte) at 24 h, 48 h, and 72 h. Data are expressed as mean ± SEM; * *p* < 0.05 and *** *p* < 0.001; the significant difference is compared to C: control cells without treatment.

In Figure 5, the results of the viability of SLN at different incubation times are observed.

MTT reduction values of untreated control cells were set as 100% cell viability. One-way ANOVA was performed, followed by the Bonferroni test for the significance of differences among multiple experimental groups. Data are expressed as mean ± SEM; * *p* < 0.05, ** *p* < 0.01, and *** *p* < 0.001; the significant difference is compared to C: control cells without treatment. Concentrations of CHON were used with values of 0.08, 0.04, and 0.016 mg/mL to treat the cells. 

The conditions established in this cell viability study show that no cell cytotoxicity was found at the established concentrations and times; while initial cytotoxicity was observed at 24 h and 48 h after treatment, at 0.04 mg/mL of CHON, the cells recovered quickly and showed almost normal viability at later treatment time points [33]. 

### 2.3. Results of Cellular Internalization of Chondroitin Sulfate Nanoparticles

A cellular uptake study was conducted to assess the capacity of chondroitin sulfate nanoparticles to enter HaCat cells. The nanoparticles were marked with Nile Red, a fluorescent dye that interacts preferentially with lipids, enabling visualization without the need for dissolution [34].

**Figure 6:**
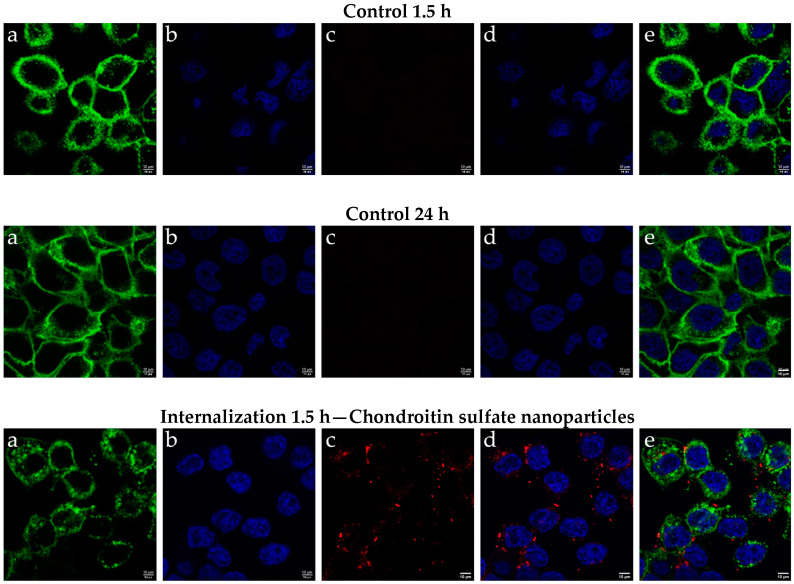

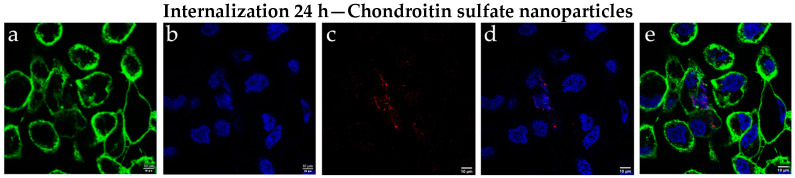
Cellular uptake by confocal microscopy analysis in HaCaT cells. Cells were incubated for 1.5 h and 24 h with the indicated Nile red chondroitin sulfate nanoparticles. (**a**) Membrane staining with WGA; (**b**) nuclei staining with DAPI; (**c**) fluorescence of internalized chondroitin sulfate nanoparticles; (**d**) merged (**b**,**c**), and (**e**) merged (**a**–**c**). The figure scale bar corresponds to 10 μm.

As illustrated in Figure 6, the internalization of nanoparticles was evaluated at non-cytotoxic concentrations at two points in time, as established in the cell viability study: at 1.5 h and 24 h post-treatment. The results indicate successful internalization at both time intervals. However, the fluorescence intensity was significantly higher at 1.5 h compared to 24 h. This observation suggests that while initial uptake is robust, there is a marked decrease in fluorescence intensity over time, likely due to endosomal processing and metabolic degradation of the nanoparticles within the cellular environment after 1.5 h.

These findings highlight the importance of timing in evaluating nanoparticle internalization and suggest that understanding the pathways involved in nanoparticle metabolism could provide information for future applications in targeted drug delivery systems.

### 2.4. Results of Biopharmaceutical Studies

The transdermal absorption of SLN of CHON was studied through an ex vivo permeation study using porcine ear skin. The pig ear skin samples were placed in the receptor compartment of Franz cells, which are highly valuable systems for ex vivo research on drug permeation through human skin [35,36,37]. The permeated amount of CHON at 24 h, expressed as a percentage of CHON encapsulated was also shown by median and range (Table 3).

Statistically significant differences between CHON permeate percentage (%) per group were evaluated. One-way ANOVA without parametric testing for the significance of differences between multiple experimental groups was performed. This was followed by the *t*-test (non-parametric test); the significance of the differences between each pair of experimental groups (*p* < 0.05) was obtained using the Mann–Whitney test. Statistically significant differences were found between the control group and the SLN group.

The CHON concentration in the SLN formulation was 0.4 mg/mL, achieving around 60% encapsulation. From that encapsulated percentage, the amount permeated through the pig ear skin was calculated. Permeation through the skin was achieved with the different formulations, compared to CHON 0.4 mg/mL in aqueous solution (Table 3). This gave the biological variability that occurs in this type of analysis, for example, in the thickness and the section of biological tissue, as well as the differences in the skin of the different animals. In some formulations, great variability is observed in the permeate percentage or accumulated percentage.

SLN is a viable option for transporting CHON through the skin. CHON has low skin penetration, given its high molecular size, and nanoparticles have proven to be an effective transport for CHON since they contain lipids that are also present in the human body, making it easier for them to cross the skin layers. In this study, a permeation study of SLN without chondroitin was performed. However, as no analytical signal was obtained for these SLNs without chondroitin, it was decided not to include them in the results table.

The SLN of CHON retention inside the skin was studied using porcine ear skin. CHON accumulative retention among porcine ear skin, expressed by median and range, is shown in Table 4.

Statistically significant differences between the accumulated amount of CHON in the skin (µg/g/cm^2^) per group were evaluated. A one-way ANOVA non-parametric test for the significance of differences among multiple experimental groups was performed. Following the *t*-test (non-parametric test), the significance of differences among each pair of experimental groups (*p* < 0.05) was obtained by the Mann–Whitney test. Statistically significant differences were found between the control group and the SLN group.

As can be seen in Table 4, the CHON retained in the skin was significantly higher compared to the CHON in aqueous solution with respect to the formulations with SLN. This indicates the capacity of the nanoparticles to form CHON depots in the skin, which will become available over time.

Thus, a completely non-invasive method was used to deliver a bio-macromolecule into the skin without using injections or abrasive procedures.

## 3. Materials and Methods

### 3.1. Materials

The excipients used for nanoparticles manufacturing are octadecylamine (Acros Organics, Geel, Belgium), chondroitin sulfate (batch: F-042903) (Bioibérica, Industrial Estate, Barcelona, Spain), stearic acid 50 vegetable grade (Merck, Darmstadt, Germany), purified water (Milli-Q A10 module, Millipore, Guyancourt, France), poloxamer 188 (Sigma-Aldrich by Merck, Saint Louis, MO, USA), and qualitative paper filters of 43–48 μm and 7–9 μm pore size (Filter-Lab^®^, Filtros Anoia, SA, Barcelona, Spain).

The materials used for the physicochemical analysis are D-glucuronic acid > 98% L14350 (Alfa Aesar by Thermo Fisher Scientific, Bremen, Germany), sodium tetraborate decahydrate B3545, sulfuric acid, carbazole C5132 (Sigma-Aldrich by Merck, Saint Louis, MO, USA), uranyl acetate 1%, and ethanol absolute 1.07017 (Sigma-Aldrich by Merck, Schnelldorf, Germany).

The line cells used are the HaCaT cell line (Cell Line Services, Eppelheim, Germany), and the reagents used for this cell cultures were obtained from Gibco (Cacavelos, Portugal). In this study, HaCaT cells from passages 12 to 17 were utilized. 

The materials used for the cell biology tests are DMEM culture media (Dulbecco’s Modified Eagle Medium, Gibco, MA, USA), phosphate-buffered saline (PBS) tablets (Invitrogen, Thornton, Australia), dimethyl sulfoxide (DMSO) (Merck, Darmstadt, Germany), molecular grade Milli-Q water (MQH_2_O): DNAse and Rnase free (Millipore, MA, USA), culture plates (Nalge Nunc International, Rochester, NY, USA), and tetrazolium bromide (MTT) (Sigma Aldrich, St. Louis, MO, USA).

The materials used for cellular internalizations are Nile red dye (EMD Millipore, Melbourne, Australia), phosphate-buffered saline (PBS) tablets (Invitrogen, Australia), DAPI (4′,6-diamino-2-phenylindole) (Invitrogen, Carlsbad, CA, USA and Sigma Aldrich, St. Louis, MO, USA), methanol solution (methanol:water 30:70%), paraformaldehyde (Merck, Darmstadt, Germany), and Alexa Fluor-488 (Molecular Probes) (Thermo Fisher Scientific; Life Technologies, Bedford, MA, USA).

The material used for the biopharmaceutical studies is a 0.05% solution of sodium lauryl sulfate (Sigma-Aldrich by Merck, Madrid, Spain).

### 3.2. Methods

#### 3.2.1. Nanoparticles Manufacturing

The SLNs with CHON were manufactured by the hot microemulsion technique. This method had been improved; the reduction time was reduced, and the filtration technique improved.

The lipid matrix was composed of stearic acid 50 vegetable grade, ranging between 35 and 40% of the formulation, and octadecylamine was the cationic lipid used as charged carrier, representing between 42 and 48% of the formulation. The aqueous matrix contained the chondroitin sulfate sodium (CHON), representing (5–11%) of the mix, and Poloxamer 188 corresponded to (7–8%) of the formulation.

Firstly, the raw materials are weighed individually.

The aqueous solution of CHON in ultrapure water was prepared. Subsequently, both the lipids of the lipid matrix and the components of the aqueous matrix were heated separately to a temperature above their melting point (80 °C). Next, they were mixed and stirred at 80 °C to obtain a hot emulsion. In accordance with the proposed experimental factorial design, several reaction times, stirring rates, and CHON concentrations were assessed. The microemulsion was then immediately dispersed into refrigerated ultrapure water at 4 °C to produce the core solidification of the CHON–SLN. Later, the emulsion was centrifuged at defined stirring rates (19,000 rpm) and reaction times (15 min). Then, the emulsion was filtered with two qualitative filter papers of 43–48 μm and 7–9 μm and stored.

#### 3.2.2. Factorial Design

**Figure 7:**
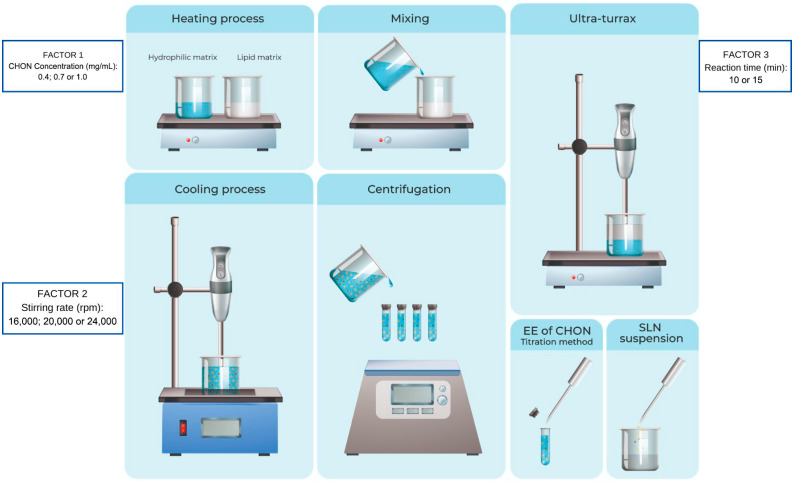
Schematic representation of solid lipid nanoparticles with CHON, prepared by the hot microemulsification process. Source: own source.

An experimental factorial design was employed to optimize the production of SLN as a CHON vehicle. A 3 × 3 × 2 full-factorial experimental design was used for the optimization of CHON–SLN formulations. The factors tested at various levels (Table 5 and Figure 7) were CHON final concentration (mg/mL) in the formulation, stirring rate during the synthesis reaction, and reaction time, which meant the time during which the synthesis reaction occurred in which it was stirred with the ultraturrax both hot and at 4 °C.

In total, the design of experiments proposed 18 different combinations (Table 6). For each experiment, two different formulations were analyzed in triplicate, and the average of the measurements for each sample was reported. Based on the results, the most optimal formulation of SLN was chosen. 

For the selection of the best formulation, different properties were tested, including the entrapment efficiency of CHON (EE%), the zeta potential, and the particle size (Table 7). The TEM technique was used to characterize the morphology and confirm the size of the nanoparticles.

Optimal factors were attained using the Minitab^®^ 18.1.0.0 software, the design of the experiment (DOE), and a Pareto chart was used in the screening step to find the significant variables.

#### 3.2.3. Physicochemical Analysis

*Morphological Analysis by Transmission Electron Microscopy (TEM)*

The morphology of the samples was evaluated using a Tecnai Spirit microscope equipped with a LaB6 cathode (FEI Company, Hillsboro, OR, USA). Images were recorded at 120 kV using a 1376 × 1024-pixel CCD Megaview camera. The samples were negatively stained with a 2.0% uranyl acetate solution and absorbed onto carbon-coated copper grids CF200-Cu.

*Determination of Surface Charge (Zeta-Potential) and Particle Size*

In the process of SLN formula optimization, analyses of SLN sizes were determined by laser diffraction on a Mastersizer 2000 (Malvern Instruments, Cambridge, UK) equipped with a 4 mW He–Ne laser (633 nm). For the treatment of samples in solution, the stirring speed was fixed at 800 rpm. The results are expressed as average surface diameter (d[3,2]) (D [3,2]) in nanometers (nm) unless otherwise indicated. The zeta potential of some formulations was also measured by laser Doppler microelectrophoresis in a Zetasizer Nano-Z (Malvern Instruments, Cambridge, UK). The zeta-potential values were obtained from the electrophoretic mobility of the nanoparticles and lipoplexes under an electric field. All samples were analyzed in triplicate, and the results are expressed as mean voltage in millivolts (mV) or nanometers (nm), depending on the measurement.

*Entrapment* *Efficiency of CHON (EE%) Determined by UV-VIS Spectroscopy Method*

The CHON concentration in the nanoparticle samples was determined using a modified method [38,39], which consisted of the acid hydrolysis of glycosaminoglycans (CHON hydrolysis in monosaccharides) [40] and the reaction of hexuronic acid with carbazole, obtaining a colorimetrically quantifiable red solution.

A total of 1 mL of the sample to be analyzed was added, either as a standard solution of glucuronic acid (100, 50, 25, and 10) mg/L, to a previously prepared solution of sodium tetraborate decahydrate or in sulfuric acid (0.08 mg/mL), in an ice-cold stoppered test tube. It was stirred and heated at 95–100 °C for 30 min. Then, it was cooled in an ice bath, and 0.20 mL of carbazole solution, prepared at a concentration of 1.25 mg/mL of absolute ethanol, was added. Subsequently, it was shaken vigorously and heated again for 30 min.

After this, it was cooled to room temperature and the final absorbance (A) of the standard solution or the samples under study was measured at 520 nm, which was compared against a blank prepared in the same way. The value of the corresponding blank was subtracted from each absorbance value. MQH_2_O was used for standard solutions, and their respective plain SLN formulation was used for SLN. The analysis of each sample was carried out in triplicate. The linear regression equation of the absorbance of the standard solutions (Ast) against their concentration (Cst mg/L) was calculated. It was calculated with the value of r 2, and the hexuronic acid content was calculated from the calibrator function, expressed as glucuronic acid in each sample. A conversion factor of 0.343 was used to pass the mg of glucuronic acid found to mg CHON of bovine origin.

% CHON = (Cmg/mL)/(initial CHONmg/mL × 0.343) × 100

#### 3.2.4. Cell Lines

The human keratinocyte (HaCaT) cell line was maintained in high-glucose Dubelcco’s Modified Eagle’s medium (DMEM) supplemented with 25 mM HEPES, 1% non-essential amino acids, 100 μg/mL streptomycin, 100 U/mL penicillin, and 10% heat inactivated fetal calf serum (FCS). Cells were cultured in a humidified incubator at 37 °C in a 5% CO_2_ atmosphere and received fresh medium every 3 days. The experiments were performed at 80–90% of confluence (passes between n = 85–95).

#### 3.2.5. Cytotoxicity Assay

The effect on cell viability of SLN containing CHON was evaluated in vitro using the MTT assay. To achieve this, 100 µL of a solution containing 1 × 10^5^ cells/mL were seeded in 96-well plates and incubated for 24 h at 37 °C in a 5% CO_2_ humidified atmosphere overnight. The media were replaced by 90 mL of fresh media plus 10 mL of media containing the formulation of nanoparticle diluted 1, 5, 10, 25, 50, and 100 times, depending on the case studied. The initial concentration of the SLN formulations was 36 µg/µL. After a period of incubation (24, 48, 72 h or 1, 3, and 5 days), cells were washed with PBS and incubated with 0.25% MTT in fresh medium for 2 h at 37 °C in the dark. Subsequently, the medium was removed; the cells were washed with PBS, and cells were lysed by the addition of 99% DMSO. Finally, the absorbance was measured at λ = 560 nm using an automatic Modulus™ Microplate Photometer (Turner BioSystems, Sunnyvale, CA, USA). The data were analyzed by calculating the percentage of MTT reduction compared to the control (untreated cells, 100% viability).

#### 3.2.6. Cellular Internalization of Chondroitin Sulfate Nanoparticles

*Characterization of Chondroitin Sulfate Nanoparticles Uptake by Confocal Microscopy*

To assess the cellular internalization of chondroitin sulfate nanoparticles, dye-labeled nanoparticles were prepared. Dye-labeled samples were created from the optimal SLN by incorporating 200 μL of a 2 mg/mL Nile red methanol solution (EMD Millipore, Melbourne, Australia) into the lipid matrix of the SLN suspension prior to the melting process [E VIGHI]. Nile red is a fluorescent dye known for its affinity for lipids, enabling their visualization without the need for dissolution [34,41].

To assess internalization of chondroitin sulfate nanoparticles, HaCaT cells, 300 µL of 1 × 10^5^ cell·mL^−1^ was grown in an 8-well chamber slider (ibidi^®^) until approximately 80% confluence and then incubated with 5 µL of nanoparticles at 37 °C for 1.5 h. Non-internalized nanoparticles were removed by three PBS washes. The plasmatic membranes of the cells were stained with wheat germ agglutinin (WGA) Alexa Fluor-488 (Molecular Probes, Oregon, USA) at 1 μg/mL for 25 min at 4 °C. The cells were washed 3 times with PBS and fixed with 3% paraformaldehyde for 30 min at 25 °C. Subsequently, the cells were washed 3 times with PBS, and the nuclei were stained with 4,6-diamino-2-phenylindole dichlorate (DAPI) at 0.5 μg/mL for 15 min at 25 °C. Finally, the cells were washed 3 times, and mounting solution (PBS) was added for microscopic analysis. Images were acquired using a Leica TCS SP5 confocal laser scanning microscope (Leica Microsystems, Mannheim, Germany) with a 63× oil immersion objective lens.

#### 3.2.7. Biopharmaceutical Studies

*Ex vivo Study of Skin Permeation–Diffusion with Franz Cells*

The transdermal absorption of 1.0 mL of SLN of CHON was estimated through an ex vivo permeation study using porcine ear skin (n = 5) with 0.6 mm thickness using Franz-type diffusion cells with a surface area of 0.64 cm^2^ and receptor chamber capacity of 4.5 mL for all the mucous membranes at 32 °C.

Specimen porcine ear skin was obtained ex vivo from female pigs (Landrace × Large White cross, 40–45 kg), following the methodology outlined by Pérez-González and colleagues [42], resulting from the surplus of surgical studies following the study Protocol of the Animal Experimentation approved by the Ethics Committee of the University of Barcelona with ethics code 514/18. Upon collection, the tissues were promptly transported to the laboratory in a solution of artificial aqueous humor for cleaning and preparation prior to experimentation [43].

Immediately, porcine ear skin was excised and debrided in the laboratory to obtain the corresponding samples of tissues and frozen by placing them in containers with a phosphate-buffered saline (PBS) mixture containing 4% albumin and 10% DMSO (as cryoprotective agents) and stored at −80 °C in a freezer until their use. The ear skin was kept flat while stored. The day before their use, partially frozen specimens were cut into 600 µm parallel slices with a mucotome GA 630 (Aesculap, Tuttlingen, Germany) (228–230), and the slices were kept frozen until use. After removing the cryoprotectant, with successive washes of PSB, slices were placed in the Franz cell holders, with the stratum corneum toward the top, which corresponds to the donor compartment (Vidra Foc, Barcelona, Spain) in which to perform the ex vivo experiment.

A total of 1 mL of the sample was placed in the donor compartment of the Franz cell, and the receptor compartment was filled with MQH_2_O at a pH of 5.92 ± 1. In all experiments, a constant temperature, thermoregulated with a water jacket of 32 ± 0.5 °C, was used, with agitation at 600 rpm. Samples were collected from the receiver compartment at 24 h. Sink conditions were maintained throughout the experiment.

To quantify the amount of CHON permeated in the tissues tested, drug concentration in the receptor phase (water) was determined by a validated technique of UV-Vis detection (520 nm) using a Thermo Scientific Helyos B Uv-Vis Dual Beam Spectrophotometer, Eindhoven, The Netherlands. 

The validation range was from 0.025 to 0.808 mg/mL of CHON in the sample, with an R^2^ greater than 0.9995, an accuracy exceeding 95.6%, and a precision greater than 95.1%, in accordance with AOAC guidelines [44,45]. Transepidermal water loss values were used for skin integrity testing. The Tewameter TM Hex probe was used to assess tissue integrity. Before placing the samples in the Franz cells, the probe was positioned to evaluate and ensure that there were no holes or injuries in the skin. Normal TEWL rates were considered to be the values of less than 15 g/(m^2^ h) ± 2 [46]. All the experiments were performed at least in quintuplicate; the results were also shown by median and range.

*Drug Retention inside the Skin*

At the end of this experiment, porcine ear skins were removed and cut into permeated areas corresponding to 0.64 cm^2^; then, they were carefully cleaned using a gauze soaked in a 0.05% solution of sodium lauryl sulfate, washed with distilled water, and blotted dry with filter paper (FILTER-LAB^®^, Filtros ANOIA, S.A. Barcelona, Spain). Then, the skin was weighed and divided into smaller pieces; 1.2 mL of MQH_2_O water was added; it was sonicated for 20 min; then, it was centrifuged for 15 min at 12,000 rpm, and the supernatant was used for the analysis of the amount of CHON retained on the skin. The amount of CP retained in each sample of skin (Qr, µg/cm^2^/g) was estimated by a validated technique of UV-Vis detection (520 nm) using a Thermo Scientific Helyos B Uv-Vis Dual Beam Spectrophotometer. Transepidermal water loss values were used for skin integrity testing. All the experiments were performed in triplicate. Again, the results are shown in terms of median and range.

## 4. Conclusions

The production of solid lipid nanoparticles (SLN) of CHON has been optimized according to a DOE, and the factors CHON concentration of 0.4 mg/mL, stirring rates of 20,000 rpm, and a reaction time of 10 min have been established as optimal. The morphology of the nanoparticles analyzed through transmission electron microscopy (TEM) reveals that the nanoparticles show spherical morphologies. The average sizes remained around 150 nm, and the zeta potential values were around −40 mV. It has been demonstrated that SLN formulations had the ability to internalize in HaCaT cells. The optimal concentration for the cell viability study has been determined. It was established that CHON concentrations in the range of 0.08 mg/mL to 0.016 mg/mL do not cause cytotoxicity. Cell internalization has been demonstrated at 1.5 h and 24 h post-treatment. It has been verified that SLN formulations are an effective vehicle for the transport of CHON through the skin of pig ears, showing a greater permeation of CHON in the nanoparticle compared to CHON in an aqueous medium. SLN retention studies show that CHON retained on the skin is significantly higher when comparing CHON in aqueous solution to SLN formulations. These results indicate the capacity of the nanoparticles to form depots of Chon in the skin, which will become available over time.

## Figures and Tables

**Table 1:** Experimental factorial design (3 × 3 × 2) for the optimization of the formulation of SLN-1 and the results obtained for each experiment analyzed.

Experiment	Concentration (mg/mL)	Stirring Rate (rpm)	Reaction Time (min)	Entrapment Efficiency (EE %)		Z Potential (mV)		Particle Size (nm)		Particle Size
				Mean	SD	Mean	SD	Mean	SD	Johnson’s Transformation ^(1)^
1	0.4	16,000	10	48.24	1.21	−45.67	0.98	155.33	2.08	1.62853
2	0.4	16,000	15	52.77	1.48	−41.67	0.72	136.67	0.58	0.97257
3	0.4	20,000	10	59.57	1.27	−39.10	1.61	118.50	0.71	−0.30277
4	0.4	20,000	15	55.16	0.55	−38.17	1.29	115.50	0.71	−0.71042
5	0.4	24,000	10	56.80	0.00	−39.43	0.23	121.00	2.00	−0.03776
6	0.4	24,000	15	53.37	0.02	−40.13	1.70	180.00	11.31	1.69187
7	0.7	16,000	10	35.54	1.29	−47.53	1.10	147.50	0.71	2.01961
8	0.7	16,000	15	44.27	0.00	−46.83	0.97	143.00	1.41	1.23266
9	0.7	20,000	10	45.74	0.97	−41.90	0.44	123.00	0.00	0.14203
10	0.7	20,000	15	33.15	0.05	−40.33	0.15	120.00	1.73	−0.13758
11	0.7	24,000	10	30.34	0.06	−38.13	0.61	121.67	12.50	0.02488
12	0.7	24,000	15	31.86	0.00	−44.83	0.40	121.50	2.52	0.00949
13	1.0	16,000	10	22.67	0.62	−50.97	0.64	161.00	4.36	1.77747
14	1.0	16,000	15	17.41	0.62	−48.03	1.40	127.00	8.66	0.44232
15	1.0	20,000	10	25.44	0.00	−48.47	0.61	111.67	0.58	−1.52134
16	1.0	20,000	15	22.23	0.00	−44.67	0.45	130.00	8.19	0.63032
17	1.0	24,000	10	26.18	0.44	−49.73	0.90	129.67	7.51	0.61069
18	1.0	24,000	15	41.17	0.03	−51.23	0.67	122.33	0.58	0.08473
19	0.4	16,000	10	48.25	0.00	−36.63	0.31	139.00	2.83	1.07435
20	0.4	16,000	15	59.85	1.49	−40.37	0.81	112.00	0.00	−1.42783
21	0.4	20,000	10	63.01	0.00	−29.53	0.51	117.33	3.79	−0.44696
22	0.4	20,000	15	51.38	0.60	−39.75	0.35	112.33	2.31	−1.34042
23	0.4	24,000	10	52.77	1.05	−35.87	0.95	112.00	2.00	−1.42783
24	0.4	24,000	15	55.91	0.00	−40.00	0.53	116.00	3.46	−0.63329
25	0.7	16,000	10	30.80	0.00	−41.47	1.53	120.00	0.00	−0.13758
26	0.7	16,000	15	39.99	0.44	−43.93	1.56	125.00	2.65	0.30055
27	0.7	20,000	10	48.56	0.02	−46.03	0.86	130.00	1.41	0.63032
28	0.7	20,000	15	33.85	0.36	−46.77	0.40	109.00	2.83	−2.67674
29	0.7	24,000	10	29.48	0.72	−47.57	1.43	124.67	0.58	0.27540
30	0.7	24,000	15	32.00	0.00	−44.37	0.15	117.67	3.21	−0.40416
31	1.0	16,000	10	23.83	0.30	−51.70	0.50	143.67	0.58	1.25732
32	1.0	16,000	15	18.73	0.00	−46.93	1.90	117.33	3.06	−0.44696
33	1.0	20,000	10	25.27	0.29	−49.13	0.78	110.67	0.58	−1.84872
34	1.0	20,000	15	19.83	0.85	−46.33	0.87	112.50	0.50	−1.29876
35	1.0	24,000	10	25.31	0.00	−51.70	0.36	116.00	0.00	−0.63329
36	1.0	24,000	15	39.77	0.88	−47.07	0.71	117.33	2.31	−0.44696

^(1)^ In the case of the evaluation of the size values, a Johnson transformation was required.

**Table 2:** Factors and results of the best formulation obtained through the DOE.

CHON Concentration (mg/mL)	Stirring Rate (rpm)	Reaction Time (min)	EE %		Pot Z (mV)		Size (nm)	
			Mean	SD	Mean	SD	Mean	SD
0.4	20,000	10	61.29	2.05	−34.32	5.35	117.80	2.77

**Table 3:** CHON skin permeation results expressed in mg of CHON and percentage through an ex vivo permeation study using porcine ear skin.

Sample	CHON Cumulative Permeate Amount (mg)	Percentage Permeates of CHON (%)	Median and Range (%)
Control (CHON 0.4 mg/mL in aqueous solution)	0.0060	1.47	2.44 (1.47–3.41)
	0.0140	3.41	
	0.0070	1.82	
	0.0120	3.06	
	0.0070	1.66	
	0.0130	3.22	
SLN	0.2119	86.98	77.03 (58.4–86.98)
	0.1968	84.96	
	0.1784	77.03	
	0.1353	58.40	
	0.1661	71.70	

**Table 4:** CHON accumulative retention among porcine ear skin.

Sample	Amount Retained in Skin (Qr, µg/g/cm^2^)	Median and Range
Control (CHON 0.4 mg/mL in aqueous solution)	4.51	6.67 (4.49–8.83)
	8.83	
	4.49	
	8.82	
SLN-1	173.20	499.80 (173.20–567.01)
	435.46	
	567.01	
	564.05	

**Table 5:** Factors and levels used in the experimental factorial design to optimize the production of SLN as a vehicle of CHON.

Factors	Levels
Concentration (mg/mL)	0.4–0.7–1.0
Stirring rate (rpm)	16,000–20,000–24,000
Reaction time (min)	10–15

**Table 6:** Design of experiments to optimize the production of SLN as a vehicle of CHON.

Experiment	Concentration (mg/mL)	Stirring Speed (rpm)	Reaction Time (min)
1	0.4	16,000	10
2	0.4	16,000	15
3	0.4	20,000	10
4	0.4	20,000	15
5	0.4	24,000	10
6	0.4	24,000	15
7	0.7	16,000	10
8	0.7	16,000	15
9	0.7	20,000	10
10	0.7	20,000	15
11	0.7	24,000	10
12	0.7	24,000	15
13	1.0	16,000	10
14	1.0	16,000	15
15	1.0	20,000	10
16	1.0	20,000	15
17	1.0	24,000	10
18	1.0	24,000	15

**Table 7:** Properties evaluated to optimize the production of SLN as a vehicle of CHON.

Property	Criteria
Entrapment efficiency of CHON (EE%)	Highest % EE possible
Zeta potential (mV)	<−25 and >25
Particle size (nm)	Minor size possible
Morphology	TEM technique to characterize SLN

## Data Availability

The data presented in this study are available upon request to the corresponding author due to confidentiality issues.

## Supplementary Materials

The following supporting information can be downloaded at https://www.mdpi.com/article/10.3390/ijms251810023/s1.
