# Supplementary material for: Chondroitin Sulfate for Cartilage Regeneration, Administered Topically Using a Nanostructured Formulation
Source: Int J Mol Sci. 2024 Sep 18;25(18):10023. doi: 10.3390/ijms251810023 (PMC11432425; doi:10.3390/ijms251810023)
Supplement: Supplementary file 1 [file ijms-25-10023-s001.zip › ijms-3153601-supplementary.pdf]

International Journal of Molecular Sciences

MDPI

Supplementary material 1 (S1)

ANALYSIS OF DATA- EXPERIMENTAL FACTORIAL DESIGN

The Data was attained using the Minitab® 18.1.0.0 software.

Evaluation of statistical premises

In the first place, the statistical assumptions were evaluated for model validity. The obtained data was assessed to confirm a normal distribution for the three evaluated outcomes:

i. Entrapment efficiency (EE %)
ii. Z Potential (mV)
iii. Particle Size (nm)

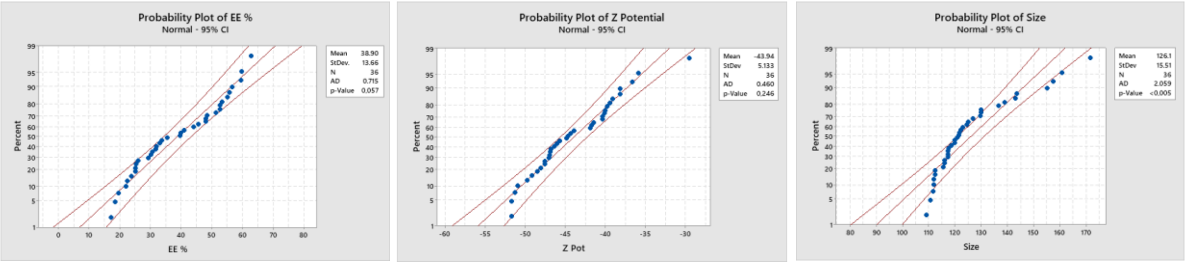

Figure S1: Graph for Normality Assessment of Data, with a p-value of ≥0.05.

As observed in Figure S1, the data obtained for the EE % and Z potential percentage follows a normal distribution, p value is greater than 0.05, follows normal distribution. However, the data collected for the nanoparticle size does not follow a normal distribution, p value less than 0.05: does not follow normal distribution.

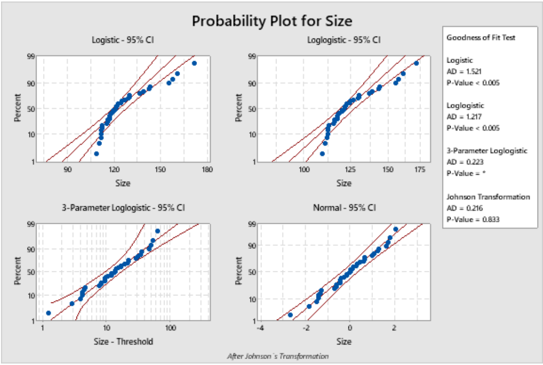

Figure S2: Probability Plot for Size Transformed Data

Johnson transformation is effective and the normal distribution is a good fit for the transformed data, the points on the plot for the transformed data closely follow the fitted normal distribution line with p value greater than 0.05 for the Johnson distribution.

International Journal of *Molecular Sciences*

MDPI

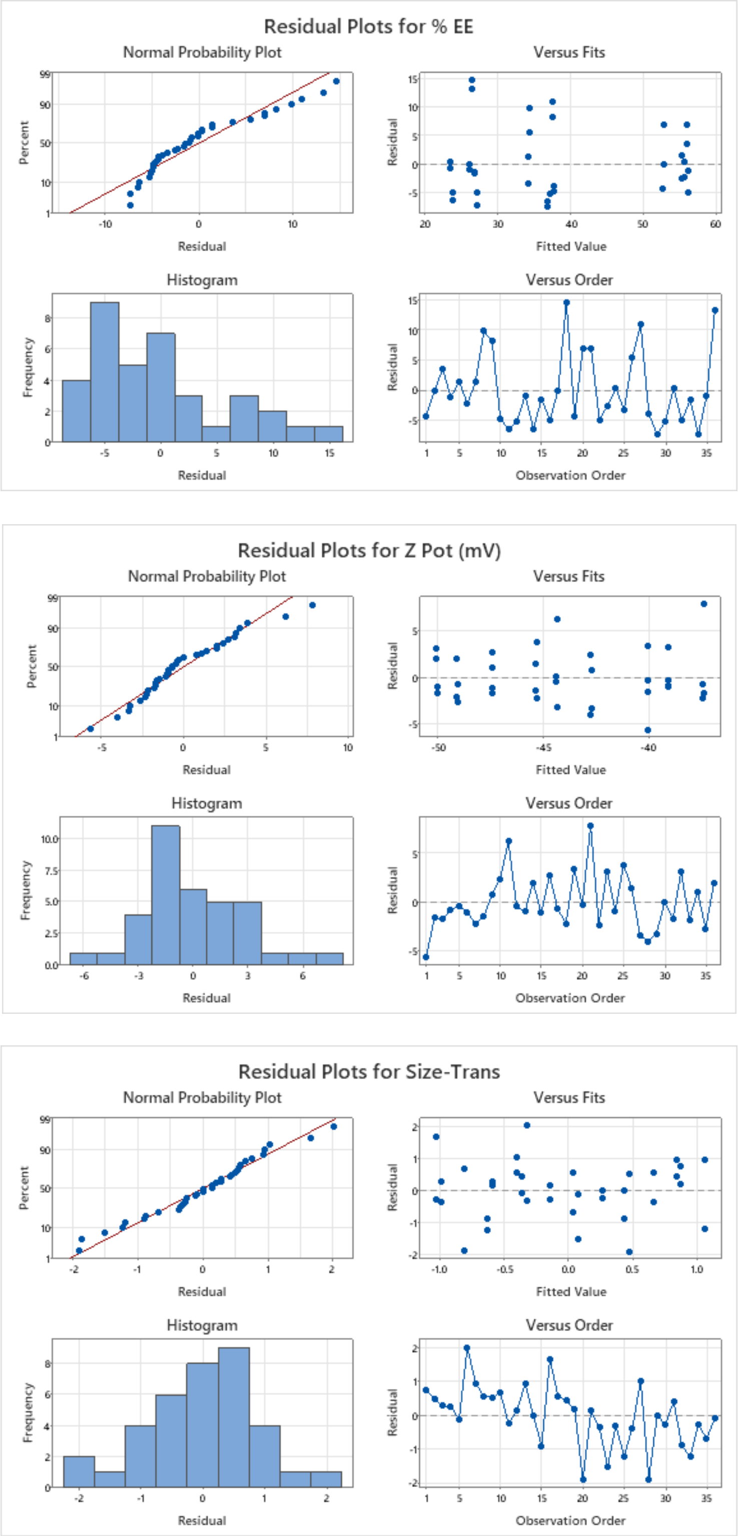

Figure S3: Plots of the DOE premises evaluation.

International Journal of Molecular Sciences

MDPI

According to Figure S3, the following results are obtained when analyzing the assumptions for the use of a Design of Experiments (DOE).

1. *Residual normality:* all the result values are aligned to the red line.
2. *Independence:* residuals vs. order of observation, there is no attention - grabbing order: some pattern above or below . There is independence.
3. *Homoscedasticity:* second graph on the upper right. The residues are painted. The fitted value of the residuals shows values above and below zero.

Analysis of variance for Analyze Factorial Design, p-values for the two-way interactions

i. Entrapment efficiency (% EE)

Table S1. Factor Information in the Analysis of variance of % EE

Factor	Levels	Values
Concentration (mg/ml)	3	0.4, 0.7, 1.0
Stirring Rate (rpm)	3	16000, 20000, 24000
Reaction Time (min)	2	10, 15

The table S1 shows the factors and levels for each factor, used in the Analysis of variance.

Table S2. Analysis of Variance for the response % EE

Source	DF	Seq SS	Contribution	Adj SS	Adj MS
Model	13	6162.53	94.36%	6162.53	474.04
Linear	5	5282.12	80.88%	5282.12	1056.42
Concentration (mg/ml)	2	5203.61	79.68%	5203.61	2601.81
Stirring Rate (rpm)	2	77.84	1.19%	77.84	38.92
Reaction Time (min)	1	0.66	0.01%	0.66	0.66
2-Way Interactions	8	880.41	13.48%	880.41	110.05
Concentration (mg/ml)*Stirring Rate (rpm)	4	506.35	7.75%	506.35	126.59
Concentration (mg/ml)*Reaction Time (min)	2	10.80	0.17%	10.80	5.40
Stirring Rate (rpm)*Reaction Time (min)	2	363.27	5.56%	363.27	181.63
Error	22	368.11	5.64%	368.11	16.73
Lack-of-Fit	4	287.84	4.41%	287.84	71.96
Pure Error	18	80.27	1.23%	80.27	4.46
Total	35	6530.64	100.00%		

Source	F-Value	P-Value
Model	28.33	0.000
Linear	63.14	0.000
Concentration (mg/ml)	155.50	0.000
Stirring Rate (rpm)	2.33	0.121
Reaction Time (min)	0.04	0.844
2-Way Interactions	6.58	0.000
Concentration (mg/ml)*Stirring Rate (rpm)	7.57	0.001
Concentration (mg/ml)*Reaction Time (min)	0.32	0.728
Stirring Rate (rpm)*Reaction Time (min)	10.86	0.001
Error		
Lack-of-Fit	16.14	0.000

Pure Error	
Total	

Table S3. Model Summary

S	R-sq	R-sq(adj)	PRESS	R-sq(pred)	AICc	BIC
4.09052	94.36%	91.03%	985.686	84.91%	239.86	239.61

According to Table S3, the proposed model predicts over 90% of the variation in the results obtained.

Table S4. Coefficients table for Analyze Factorial Design for %EE

Term	Coef	SE Coef	95% CI	T-Value
Constant	38.903	0.682	(37.489, 40.317)	57.06
Concentration (mg/ml)				
0.4	15.853	0.964	(13.853, 17.852)	16.44
0.7	-2.604	0.964	(-4.603, -0.604)	-2.70
Stirring Rate (rpm)				
16000	-2.042	0.964	(-4.041, -0.042)	-2.12
20000	1.364	0.964	(-0.636, 3.363)	1.41
Reaction Time (min)				
10	-0.136	0.682	(-1.550, 1.278)	-0.20
Concentration (mg/ml)*Stirring Rate (rpm)				
0.4 16000	-0.44	1.36	(-3.27, 2.39)	-0.32
0.4 20000	1.16	1.36	(-1.67, 3.99)	0.85
0.7 16000	3.39	1.36	(0.56, 6.22)	2.49
0.7 20000	2.66	1.36	(-0.16, 5.49)	1.95
Concentration (mg/ml)*Reaction Time (min)				
0.4 10	0.153	0.964	(-1.847, 2.152)	0.16
0.7 10	0.581	0.964	(-1.418, 2.581)	0.60
Stirring Rate (rpm)*Reaction Time (min)				
16000 10	-1.838	0.964	(-3.837, 0.162)	-1.91
20000 10	4.469	0.964	(2.469, 6.468)	4.64

Term	P-Value	VIF
Constant	0.000	
Concentration (mg/ml)		
0.4	0.000	1.33
0.7	0.013	1.33
Stirring Rate (rpm)		
16000	0.046	1.33
20000	0.171	1.33
Reaction Time (min)		
10	0.844	1.00
Concentration (mg/ml)*Stirring Rate (rpm)		
0.4 16000	0.750	1.78
0.4 20000	0.404	1.78
0.7 16000	0.021	1.78
0.7 20000	0.064	1.78
Concentration (mg/ml)*Reaction Time (min)		
0.4 10	0.876	1.33
0.7 10	0.553	1.33
Stirring Rate (rpm)*Reaction Time (min)		
16000 10	0.070	1.33
20000 10	0.000	1.33

The P-value ≤ 0.05 in the table S4, implies that there is association between the term and the response.

Regression Equation

The regression equation that describes the relationship between the response % EE and the terms in the model is:

% EE = 38.903 + 15.853 Concentration (mg/ml)_0.4 - 2.604 Concentration (mg/ml)_0.7
- 13.249 Concentration (mg/ml)_1.0 - 2.042 Stirring Rate (rpm)_16000
+ 1.364 Stirring Rate (rpm)_20000 + 0.678 Stirring Rate (rpm)_24000
- 0.136 Reaction Time (min)_10 + 0.136 Reaction Time (min)_15
- 0.44 Concentration (mg/ml)*Stirring Rate (rpm)_0.4 16000
+ 1.16 Concentration (mg/ml)*Stirring Rate (rpm)_0.4 20000
- 0.72 Concentration (mg/ml)*Stirring Rate (rpm)_0.4 24000
+ 3.39 Concentration (mg/ml)*Stirring Rate (rpm)_0.7 16000
+ 2.66 Concentration (mg/ml)*Stirring Rate (rpm)_0.7 20000
- 6.05 Concentration (mg/ml)*Stirring Rate (rpm)_0.7 24000
- 2.95 Concentration (mg/ml)*Stirring Rate (rpm)_1.0 16000
- 3.82 Concentration (mg/ml)*Stirring Rate (rpm)_1.0 20000
+ 6.78 Concentration (mg/ml)*Stirring Rate (rpm)_1.0 24000
+ 0.153 Concentration (mg/ml)*Reaction Time (min)_0.4 10
- 0.153 Concentration (mg/ml)*Reaction Time (min)_0.4 15
+ 0.581 Concentration (mg/ml)*Reaction Time (min)_0.7 10
- 0.581 Concentration (mg/ml)*Reaction Time (min)_0.7 15
- 0.734 Concentration (mg/ml)*Reaction Time (min)_1.0 10
+ 0.734 Concentration (mg/ml)*Reaction Time (min)_1.0 15
- 1.838 Stirring Rate (rpm)*Reaction Time (min)_16000 10
+ 1.838 Stirring Rate (rpm)*Reaction Time (min)_16000 15
+ 4.469 Stirring Rate (rpm)*Reaction Time (min)_20000 10
- 4.469 Stirring Rate (rpm)*Reaction Time (min)_20000 15
- 2.631 Stirring Rate (rpm)*Reaction Time (min)_24000 10
+ 2.631 Stirring Rate (rpm)*Reaction Time (min)_24000 15

ii. *Z Potential (mV)*

Table S5. Factor Information in the Analysis of variance of Z Potential

Factor	Levels	Values
Concentration (mg/ml)	3	0.4, 0.7, 1.0
Stirring Rate (rpm)	3	16000, 20000, 24000
Reaction Time (min)	2	10, 15

Table S6. Analysis of Variance for the response Z Potential

Source	DF	Seq SS	Contribution	Adj SS	Adj MS
Model	13	706.750	76.63%	706.750	54.365
Linear	5	639.641	69.36%	639.641	127.928
Concentration (mg/ml)	2	597.209	64.76%	597.209	298.604
Stirring Rate (rpm)	2	42.413	4.60%	42.413	21.206
Reaction Time (min)	1	0.020	0.00%	0.020	0.020
2-Way Interactions	8	67.109	7.28%	67.109	8.389
Concentration (mg/ml)*Stirring Rate (rpm)	4	18.457	2.00%	18.457	4.614
Concentration (mg/ml)*Reaction Time (min)	2	42.930	4.65%	42.930	21.465
Stirring Rate (rpm)*Reaction Time (min)	2	5.722	0.62%	5.722	2.861
Error	22	215.489	23.37%	215.489	9.795
Lack-of-Fit	4	10.915	1.18%	10.915	2.729

Pure Error	18	204.575	22.18%	204.575	11.365
Total	35	922.240	100.00%		

Source	F-Value	P-Value
Model	5.55	0.000
Linear	13.06	0.000
Concentration (mg/ml)	30.49	0.000
Stirring Rate (rpm)	2.17	0.139
Reaction Time (min)	0.00	0.964
2-Way Interactions	0.86	0.566
Concentration (mg/ml)*Stirring Rate (rpm)	0.47	0.756
Concentration (mg/ml)*Reaction Time (min)	2.19	0.136
Stirring Rate (rpm)*Reaction Time (min)	0.29	0.750
Error		
Lack-of-Fit	0.24	0.912
Pure Error		
Total		

Table S7. Model Summary

S	R-sq	R-sq(adj)	PRESS	R-sq(pred)	AICc	BIC
3.12969	76.63%	62.83%	577.013	37.43%	220.58	220.33

According to Table 7, the proposed model predicts over 70% of the variation in the results obtained.

Table S8. Coefficients table for Analyze Factorial Design for Z Potential

Term	Coef	SE Coef	95% CI	T-Value
Constant	- 43.944	0.522	(-45.026, -42.862)	-84.25
Concentration (mg/ml)				
0.4	5.084	0.738	(3.554, 6.614)	6.89
0.7	-0.198	0.738	(-1.728, 1.332)	-0.27
Stirring Rate (rpm)				
16000	-1.200	0.738	(-2.730, 0.329)	-1.63
20000	1.429	0.738	(-0.101, 2.959)	1.94
Reaction Time (min)				
10	0.024	0.522	(-1.058, 1.105)	0.05
Concentration (mg/ml)*Stirring Rate (rpm)				
0.4 16000	-1.02	1.04	(-3.19, 1.14)	-0.98
0.4 20000	0.79	1.04	(-1.37, 2.96)	0.76
0.7 16000	0.40	1.04	(-1.76, 2.56)	0.38
0.7 20000	-1.05	1.04	(-3.21, 1.12)	-1.00
Concentration (mg/ml)*Reaction Time (min)				
0.4 10	1.131	0.738	(-0.399, 2.660)	1.53
0.7 10	0.346	0.738	(-1.184, 1.876)	0.47
Stirring Rate (rpm)*Reaction Time (min)				
16000 10	-0.540	0.738	(-2.070, 0.990)	-0.73
20000 10	0.131	0.738	(-1.399, 1.660)	0.18

Term	P-Value	VIF
Constant	0.000	
Concentration (mg/ml)		
0.4	0.000	1.33
0.7	0.791	1.33
Stirring Rate (rpm)		
16000	0.118	1.33
20000	0.066	1.33

Reaction Time (min)		
10	0.964	1.00
Concentration (mg/ml)*Stirring Rate (rpm)		
0.4 16000	0.337	1.78
0.4 20000	0.455	1.78
0.7 16000	0.705	1.78
0.7 20000	0.327	1.78
Concentration (mg/ml)*Reaction Time (min)		
0.4 10	0.140	1.33
0.7 10	0.644	1.33
Stirring Rate (rpm)*Reaction Time (min)		
16000 10	0.472	1.33
20000 10	0.861	1.33

Regression Equation

The regression equation that describes the relationship between the response Z Potential and the terms in the model is:

Z Pot (mV) = -43.944 + 5.084 Concentration (mg/ml)_0.4
- 0.198 Concentration (mg/ml)_0.7
- 4.887 Concentration (mg/ml)_1.0 - 1.200 Stirring Rate (rpm)_16000
+ 1.429 Stirring Rate (rpm)_20000 - 0.228 Stirring Rate (rpm)_24000
+ 0.024 Reaction Time (min)_10 - 0.024 Reaction Time (min)_15
- 1.02 Concentration (mg/ml)*Stirring Rate (rpm)_0.4 16000
+ 0.79 Concentration (mg/ml)*Stirring Rate (rpm)_0.4 20000
+ 0.23 Concentration (mg/ml)*Stirring Rate (rpm)_0.4 24000
+ 0.40 Concentration (mg/ml)*Stirring Rate (rpm)_0.7 16000
- 1.05 Concentration (mg/ml)*Stirring Rate (rpm)_0.7 20000
+ 0.64 Concentration (mg/ml)*Stirring Rate (rpm)_0.7 24000
+ 0.62 Concentration (mg/ml)*Stirring Rate (rpm)_1.0 16000
+ 0.25 Concentration (mg/ml)*Stirring Rate (rpm)_1.0 20000
- 0.87 Concentration (mg/ml)*Stirring Rate (rpm)_1.0 24000
+ 1.131 Concentration (mg/ml)*Reaction Time (min)_0.4 10
- 1.131 Concentration (mg/ml)*Reaction Time (min)_0.4 15
+ 0.346 Concentration (mg/ml)*Reaction Time (min)_0.7 10
- 0.346 Concentration (mg/ml)*Reaction Time (min)_0.7 15
- 1.476 Concentration (mg/ml)*Reaction Time (min)_1.0 10
+ 1.476 Concentration (mg/ml)*Reaction Time (min)_1.0 15
- 0.540 Stirring Rate (rpm)*Reaction Time (min)_16000 10
+ 0.540 Stirring Rate (rpm)*Reaction Time (min)_16000 15
+ 0.131 Stirring Rate (rpm)*Reaction Time (min)_20000 10
- 0.131 Stirring Rate (rpm)*Reaction Time (min)_20000 15
+ 0.410 Stirring Rate (rpm)*Reaction Time (min)_24000 10
- 0.410 Stirring Rate (rpm)*Reaction Time (min)_24000 15

Table S9. Fits and Diagnostics for Unusual Observations

Obs	Z Pot (mV)	Fit	SE Fit	95% CI	Resid	Std Resid	Del Resid	HI
1	-45.67	-40.47	1.95	(-44.52, -36.42)	-5.20	-2.12	-2.33	0.388889
21	-29.53	-35.35	1.95	(-39.40, -31.31)	5.82	2.38	2.70	0.388889

Obs	Cook's D	DFITS	
1	0.21	-1.85698	R
21	0.26	2.15093	R

R Large residual

International Journal of *Molecular Sciences*

MDPI

iii. *Particle Size (nm)*

Table S10. Factor Information in the Analysis of variance of Particle Size

Factor	Levels	Values
Concentration (mg/ml)	3	0.4, 0.7, 1.0
Stirring Rate (rpm)	3	16000, 20000, 24000
Reaction Time (min)	2	10, 15

Table S11. Analysis of Variance for the response Particle Size

Source	DF	Seq SS	Contribution	Adj SS	Adj MS
Model	13	18.4378	43.76%	18.4378	1.41829
Linear	5	14.7035	34.89%	14.7035	2.94070
Concentration (mg/ml)	2	0.3428	0.81%	0.3428	0.17139
Stirring Rate (rpm)	2	12.9035	30.62%	12.9035	6.45175
Reaction Time (min)	1	1.4572	3.46%	1.4572	1.45723
2-Way Interactions	8	3.7343	8.86%	3.7343	0.46679
Concentration (mg/ml)*Stirring Rate (rpm)	4	0.3573	0.85%	0.3573	0.08932
Concentration (mg/ml)*Reaction Time (min)	2	0.6798	1.61%	0.6798	0.33990
Stirring Rate (rpm)*Reaction Time (min)	2	2.6972	6.40%	2.6972	1.34861
Error	22	23.6993	56.24%	23.6993	1.07724
Lack-of-Fit	4	7.2056	17.10%	7.2056	1.80139
Pure Error	18	16.4937	39.14%	16.4937	0.91632
Total	35	42.1371	100.00%		

Source	F-Value	P-Value
Model	1.32	0.275
Linear	2.73	0.046
Concentration (mg/ml)	0.16	0.854
Stirring Rate (rpm)	5.99	0.008
Reaction Time (min)	1.35	0.257
2-Way Interactions	0.43	0.888
Concentration (mg/ml)*Stirring Rate (rpm)	0.08	0.987
Concentration (mg/ml)*Reaction Time (min)	0.32	0.733
Stirring Rate (rpm)*Reaction Time (min)	1.25	0.306
Error		
Lack-of-Fit	1.97	0.143
Pure Error		
Total		

Table S12. Model Summary

S	R-sq	R-sq(adj)	PRESS	R-sq(pred)	AICc	BIC
1.03790	43.76%	10.52%	63.4592	0.00%	141.11	140.87

According to Table S12, the proposed model predicts over 40 % of the variation in the results obtained.

International Journal of *Molecular Sciences*

MDPI

Table S13. Coefficients table for Analyze Factorial Design for Particle Size

Term	Coef	SE Coef	95% CI	T-Value
Constant	-0.030	0.173	(-0.389, 0.329)	-0.17
Concentration (mg/ml)				
0.4	-0.050	0.245	(-0.558, 0.457)	-0.21
0.7	0.136	0.245	(-0.371, 0.644)	0.56
Stirring Rate (rpm)				
16000	0.754	0.245	(0.247, 1.262)	3.08
20000	-0.710	0.245	(-1.218, -0.203)	-2.90
Reaction Time (min)				
10	0.201	0.173	(-0.158, 0.560)	1.16
Concentration (mg/ml)*Stirring Rate (rpm)				
0.4 16000	-0.112	0.346	(-0.830, 0.605)	-0.32
0.4 20000	0.090	0.346	(-0.627, 0.808)	0.26
0.7 16000	-0.007	0.346	(-0.725, 0.710)	-0.02
0.7 20000	0.093	0.346	(-0.624, 0.811)	0.27
Concentration (mg/ml)*Reaction Time (min)				
0.4 10	-0.040	0.245	(-0.547, 0.467)	-0.16
0.7 10	0.185	0.245	(-0.323, 0.692)	0.75
Stirring Rate (rpm)*Reaction Time (min)				
16000 10	0.344	0.245	(-0.163, 0.852)	1.41
20000 10	-0.019	0.245	(-0.526, 0.488)	-0.08

Term	P-Value	VIF
Constant	0.865	
Concentration (mg/ml)		
0.4	0.839	1.33
0.7	0.583	1.33
Stirring Rate (rpm)		
16000	0.005	1.33
20000	0.008	1.33
Reaction Time (min)		
10	0.257	1.00
Concentration (mg/ml)*Stirring Rate (rpm)		
0.4 16000	0.748	1.78
0.4 20000	0.797	1.78
0.7 16000	0.984	1.78
0.7 20000	0.790	1.78
Concentration (mg/ml)*Reaction Time (min)		
0.4 10	0.872	1.33
0.7 10	0.458	1.33
Stirring Rate (rpm)*Reaction Time (min)		
16000 10	0.173	1.33
20000 10	0.939	1.33

The P-value ≤ 0.05 in the table S13, implies that there is association between the term and the response.

Regression Equation

The regression equation that describes the relationship between the response Particle Size and the terms in the model is:

Regression Equation

Size-Trans = -0.030 - 0.050 Concentration (mg/ml)_0.4 + 0.136 Concentration (mg/ml)_0.7
- 0.086 Concentration (mg/ml)_1.0 + 0.754 Stirring Rate (rpm)_16000
- 0.710 Stirring Rate (rpm)_20000 - 0.044 Stirring Rate (rpm)_24000
+ 0.201 Reaction Time (min)_10 - 0.201 Reaction Time (min)_15
- 0.112 Concentration (mg/ml)*Stirring Rate (rpm)_0.4 16000
+ 0.090 Concentration (mg/ml)*Stirring Rate (rpm)_0.4 20000
+ 0.022 Concentration (mg/ml)*Stirring Rate (rpm)_0.4 24000
- 0.007 Concentration (mg/ml)*Stirring Rate (rpm)_0.7 16000
+ 0.093 Concentration (mg/ml)*Stirring Rate (rpm)_0.7 20000
- 0.086 Concentration (mg/ml)*Stirring Rate (rpm)_0.7 24000
+ 0.119 Concentration (mg/ml)*Stirring Rate (rpm)_1.0 16000
- 0.183 Concentration (mg/ml)*Stirring Rate (rpm)_1.0 20000
+ 0.064 Concentration (mg/ml)*Stirring Rate (rpm)_1.0 24000
- 0.040 Concentration (mg/ml)*Reaction Time (min)_0.4 10
+ 0.040 Concentration (mg/ml)*Reaction Time (min)_0.4 15
+ 0.185 Concentration (mg/ml)*Reaction Time (min)_0.7 10
- 0.185 Concentration (mg/ml)*Reaction Time (min)_0.7 15
- 0.145 Concentration (mg/ml)*Reaction Time (min)_1.0 10
+ 0.145 Concentration (mg/ml)*Reaction Time (min)_1.0 15
+ 0.344 Stirring Rate (rpm)*Reaction Time (min)_16000 10
- 0.344 Stirring Rate (rpm)*Reaction Time (min)_16000 15
- 0.019 Stirring Rate (rpm)*Reaction Time (min)_20000 10
+ 0.019 Stirring Rate (rpm)*Reaction Time (min)_20000 15
- 0.325 Stirring Rate (rpm)*Reaction Time (min)_24000 10
+ 0.325 Stirring Rate (rpm)*Reaction Time (min)_24000 15

Table S14. Fits and Diagnostics for Unusual Observations

Obs	Size-Trans	Fit	SE Fit	95% CI	Resid	Std Resid	Del Resid	HI
6	1.692	0.062	0.647	(-1.280, 1.405)	1.630	2.01	2.17	0.388889
16	0.630	- 1.047	0.647	(-2.389, 0.295)	1.677	2.07	2.25	0.388889
25	-0.138	1.584	0.647	(0.242, 2.926)	-1.722	-2.12	-2.32	0.388889
28	-2.677	- 0.877	0.647	(-2.220, 0.465)	-1.799	-2.22	-2.46	0.388889

Obs	Cook's D	DFITS	
6	0.18	1.73215	R
16	0.19	1.79504	R
25	0.20	- 1.85433	R
28	0.22	- 1.96158	R

R Large residual

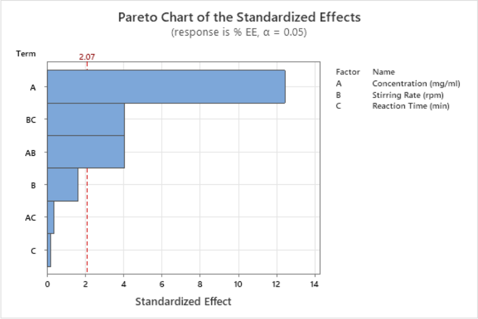

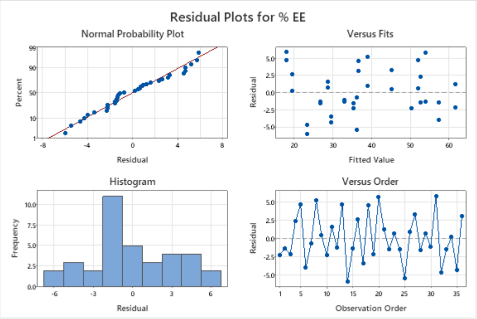

International Journal of *Molecular Sciences*

MDPI

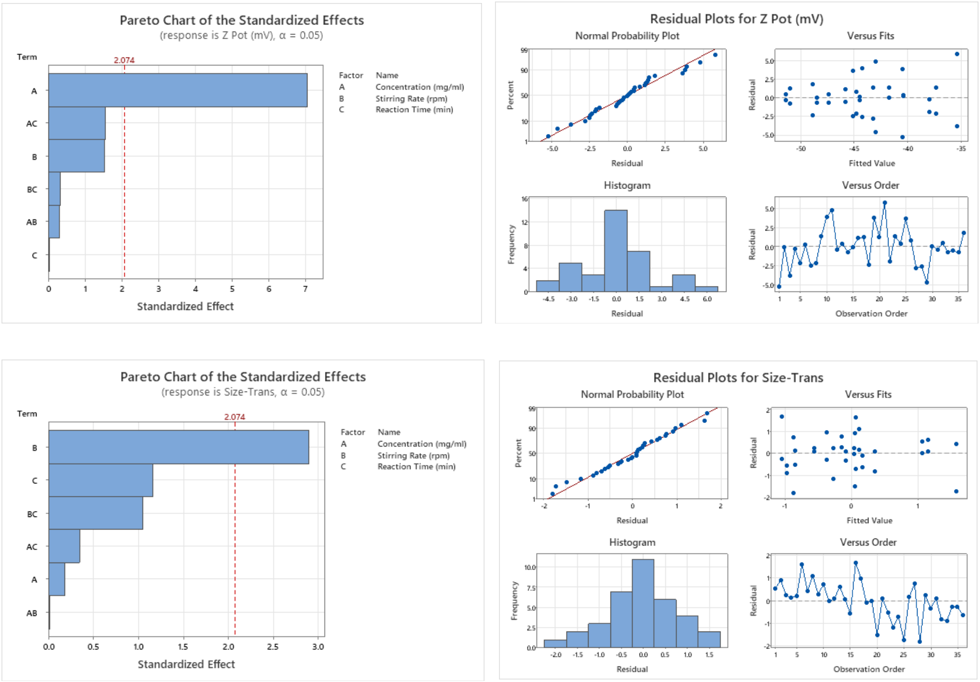

Figure S4: Pareto Charts of the Standardized effects and Residual plots, for Analysis of Variance, with p-value of ≥0.05 and considering two-way interactions

According to Figure S4, in all cases, a normal distribution of residuals is observed when considering the two-way interactions in the study. When conducting the Analysis of Variance, p-values for the two-way interactions revealed for %EE, it reveals an influence of the interaction between stirring rate and reaction time, as well as the interaction of concentration and stirring rate. In the case of Z potential, only concentration makes a significant contribution. Regarding the size, still it is the stirring rate that significantly influences, surpassing the dashed red line.
